# Assessing the extent of citrus trees root apparatus under deficit irrigation via multi-method geo-electrical imaging

**DOI:** 10.1038/s41598-019-46107-w

**Published:** 2019-07-09

**Authors:** Benjamin Mary, Daniela Vanella, Simona Consoli, Giorgio Cassiani

**Affiliations:** 1 Dipartimento di Geoscienze, Università degli Studi di Padova, Via G. Gradenigo 6, 35131 Padova, Italy; 2 Dipartimento di Agricoltura, Alimentazione e Ambiente, Università degli Studi di Catania, Via S. Sofia 100, 95128 Catania, Italy

**Keywords:** Environmental sciences, Hydrology

## Abstract

Tree rooting strategies are driven by external and internal factors such as climate conditions (rain frequency, wind direction), soil structure and crop type. In order to ensure water efficiency for irrigated crops, it is essential to know how each crop adapts its rooting strategy. We couple Mise-a-la-masse (MALM) with Electrical Resistivity Tomography (ERT) for investigating orange tree roots undergoing different irrigation strategies (Partial Root-zone Drying – or PRD - versus Full Irrigation). This is a totally novel approach giving an overall picture of roots structure and functioning in the subsoil. Our results show clear differences of rooting extent between different irrigation strategies, and identify privileged direction of root development due to distinct RWU patterns. These results are corroborated also by seasonal monitoring of evapotranspiration (ET) and soil water content (SWC), which exhibit very large differences in the soil water distribution in space and time for the trees undergoing different irrigation schedules.

## Introduction

Soil is never homogeneously explored by roots. Thus, during drought or the application of deficit irrigation (DI) regimes, soil is dried in a highly heterogeneous manner^1^ with strong variations at the decimetre scale - in dependence of the soil capillary and hydraulic properties, and of the root water uptake (RWU) processes. Indeed, the capability of soil to store and transmit water as required by roots has obvious physiological implications for plants ensuring water supply, transpiration and photosynthetic activity^2,3^. Vice versa, RWU controls the water distribution in soil. Thus it is acknowledged that the monitoring of the plant-soil-water system as a unified approach is a fairly obvious choice. However, implementing this approach in practice is not easy matter. This is the main motivation of this work. The Partial Root-Zone Drying (PRD) technique^4^ is a recent irrigation approach, capable of maintaining reasonably high yields, since crops still receive enough water from the irrigated soil zones, while fruit quality remains high, as roots enable plants to adopt water conservative strategies through hormonal signalling (Abscisic acid, or ABA) linked to the dry zones of the soil profile (e.g.^1,5^).

It must be underlined that the root structure that can be seen by excavation (e.g. using an air blade^6^) or other techniques^7^ is, in most cases, only the coarse structure, i.e. the ensemble of woody roots that give physical support to the plant. RWU is controlled by fine structures that are in connection with the woody roots, but their distribution varies as a function of depth, soil heterogeneity, and plant cover and also as a function of time, with major seasonal changes^6^. In a review article^8^ the authors describe the tree roots mechanisms in response to drought and the implication on RWU. Many studies report the effect of drought on the structure and the growth of roots with a differentiation between coarse and fine roots^9^. Unlike herbaceous plants, woody plants are characterized by extensive secondary growth, which itself can respond to drought conditions. Shao *et al*.^10^ report that water stress decreased the root length (in Acacia, Eucaliptus, groundnut), while for orange trees, Hutton *et al*.^11^ observed by soil moisture monitoring that rooting depth was very similar for both control and PRD treatment (confined in first 30–40 cm of soil profile). In a controlled environment room, Pérez-Pérez *et al*.^12^ observed that root distribution of *Citrus macrophylla* was significantly altered in PRD treatments with respect to the control treatment since the irrigated root zone had more root biomass than the drying part. They also observed that root growth was stimulated in the irrigated pot with greater biomass than in PRD. Melgar *et al*.^13^ showed that the total root length decreased in Deficit Irrigation (DI) treatment with respect to the control, but PRD did not affect any growth characteristics compared to control plants. The dry root zone of the PRD treatment had a higher specific root length, longer roots per dry weight, than the wet root zone. In addition to a growth response, there is also a molecular and anatomical response to drought that controls RWU. Mechanisms such as the reduction of the hydraulic conductivity and osmotic potential will affect the water flow, guided by the water potential gradient, in order to avoid roots dehydration. The xylem conduit (responsible for the transport) change within the plant structure. The xylem network structure and particularly its thickness is distributed to optimize the water transport from the roots to the upper part (stem, branches)^14^. Xylem conduits diameter and their walls thickness can vary to regulate the transport of water in order to cope with drought^9^.

Root system models help to establish the relations between root system architectural and hydraulic properties, and the spatio-temporal distributions of water and solute in the root zone. Warren *et al*.^15^ and Kumar *et al*.^16^ reviewed the existing mathematical models for RWU. The patterns of water uptake are strongly related to root density both in space and in time^17^. In a very instructive review paper, Dupuy *et al*.^18^ summarize the development of root growth models from its origins with simple spatial models^19,20^, while only the development of increased computational capacity has helped develop very complex plant architectural models^21^. Jourdan *et al*.^21^ advocate the use of a different approach, where roots systems are described as density distributions. The macroscopic root water models as defined by Feddes *et al*.^22^ incorporate root density distribution via an empirical relationship which has evolved with years from linear, nonlinear to exponential root distribution patterns, in all cases a function decreasing with depth. The root distribution function describes the general shapes but does not account for the real root architecture in particular in its 3D variations. Although RWU models are dependant on empirical functions describing the root density profiles, recent studies aimed to improve RWU parametrization models using observation data. Improving parameterization within models, requires introducing new components such as dynamic root distribution and root functional traits linked to resource extraction. Recently, Cai *et al*.^23^ reported an example of the application of a 3-D macroscopic RWU model considering a dynamic root distribution. Another way to improve RWU or root zone water quality models is the application of data assimilation schemes using soil moisture data^24^ or ERT^25^ data.

Although punctual, soil sampling methods are commonly used to infer root length density profiles which ultimately can be used to calibrate RWU models. The sampling depth depends on the goal of the study, but in many cases, for irrigated trees, the study the root length density is limited to the shallow subsurface (<1 m). For instance, Kadyampakeni *et al*.^26^ sampled roots only down to 30 cm depth following the recommendations of^27–29^ who demonstrated that most roots of young irrigated citrus trees in Florida (inferior or equal to three-years-old) are concentrated within 30 cm depth. Also using soil cores, Gong *et al*.^30^ attempted to better constrain RWU models using the real root density distribution of 7 years old Apple trees. The root density per cores was fitted to deduce a root density function which show a high proportions of fine roots within the top 30 cm and a root interpolated map was used to simulate RWU. For non-irrigated plants and/or specific growth conditions such as in semi-arid areas or tropical soils, the soil sampling strategy has to be adapted since root length density profiles may be very different, with much larger rooting depth. It has been shown that in a sandy tropical soil, fine roots of orange trees may reach a depth of approximately 6 m^31^. Trenches, in contrast to soil sampling, are more spatially extensive tools to infer vertical root distribution and are the most commonly adopted method in orchard research. For instance, the distribution of cherry roots was studied using the profile method in the recently published study^32^. Cherry trees seldom (average tree age of five years) reached a vertical depth of 1 m, and the main root depth at the experimental site was within the range of 0 to 80 cm. The distributions of average root length density of the cherry trees were highest at around 20 cm depth and decreased with increasing depth. In similar studies, the percentage of roots in the shallowest 10 cm of soil accounted for up to 32% of the total roots distribution of peach trees^33,34^, while for apricot and peach trees a cumulative 75–80% of roots was observed in the top 0.5 m of the soil profile. The use of water stable isotopes is also a promising approach to help calibrating RWU models. The approach has been successfully applied to apple trees, for which the root water uptake was estimated in combination with an hydrological model^35^. The results showed that the main depth of RWU ranged from 0 to 60 cm depth during the growing season, with the main contribution occurring in the 0–40 cm depth range.

Overall, the most important lesson from the previous studies is that root length density profiles are site-specific, and depend on species and water application. Consequently, the rate and the spatial distribution of root water uptake varies significantly, depending on soil water availability^13,26^, the distance from the tree trunk^34,36^, the tree age^37^, the intensity of meteorological factors during the day^32^, but also is different in the growing season^6,38^ and depends on soil practices^39^. Finally, root length density distributions may be estimated from dynamic soil moisture measurements^40,41^. For instance, Koumanov *et al*.^42^ inferred root distribution and plant water uptake from moisture probes and tensiometers on almond trees: they did not measure much root activity below the top 30 cm. In spite of technological advances in sensor development (e.g. TDR and FDR), soil water monitoring is still a challenging task, as root distribution is generally unknown and therefore it is difficult to understand the spatial distribution of RWU^43^, and thus to plan the correct location to get “representative” soil moisture values (whatever representative may mean). The main weakness of single sensors is that these soil water measurements are obtained only pointwise. The measuring volume is very small, and even when the number of probes is increased, generally no information on the lateral variation of soil water is obtained, and only a vertical profile of soil moisture is identified by placing several probes at the side of a trench, that is then backfilled. Moreover, the number of such devices cannot be increased indefinitely without serious disruption of the soil system (due to excavation) and reaching prohibitive costs (e.g.^44^). In a nutshell, point sampling of soil water content (SWC) often violates the sampling theorem, thus leading to spatial aliasing and inevitable misunderstanding of phenomena^45^. Geophysical imaging techniques, which are rapid, cost effective and practically non-invasive, allow for a wide spatial coverage and appropriate spatial resolution, thus preventing spatial aliasing problems. These techniques have been widely used as a good proxy for spatializing soil water measurements, estimating hydrogeological properties, identifying processes relevant to soil^46–52^. It is acknowledged that the electrical resistivity of a material depends on several parameters. Electrical resistivity is particularly sensitive to the distribution and the size of the soil particles but also to the soil moisture content and the electrical resistivity of the pore fluid. High resistivity, in general, means low soil moisture. The connection between electrical resistivity and soil moisture content is well established since the 1940s^53^ and with the emergence of Hydrogeophysics^54^. Electrical methods, and particularly Electrical Resistivity Tomography (ERT), have been used for studying the root biomass (e.g.^55^) and root water uptake processes (RWU) (e.g.^3,44,56^), since the root density and the resulting SWC correlate with the soil bulk electrical resistivity (e.g.^57^). Vanella *et al*.^3^ showed that, at the same study site we consider in this paper, the pattern of high electrical resistivity was controlled by soil drying caused by root water uptake and not by the roots lignified structures^55^.

The Mise-Ã -la-Masse (MALM) method is a technique also based on direct current injection, originally developed for ore deposit exploration (e.g.^58^): an electrical current is injected in a conductive body and the resulting voltage is measured at the ground surface or in boreholes: the resulting voltage contours help delineate the geometry (shape, extent, dip, continuity) of the electrically conductive target. Note that MALM can be used also for hydrogeological purposes in tracer test data (see e.g.^59^, and references therein). In the context of root imaging, the underlying assumption on the current pathway is that the current enters via the tree stem, propagates through the root system and is released in the soil only at the points where roots and soil get in contact via hair roots^60^. Thus we assume that the locations where roots extract water from the soil are the same where electrical current in the MALM configuration flow from the roots to the soil. A direct implication is that a quantitative information about RWU should only be derived from ERT, not from the MALM method that points directly towards the locations of active roots. This assumption is supported by the evidence in Mary *et al*.^60^, where injection in the stem and in the soil just next to the stem produced very different voltage patterns: in this work we adopted again this twofold approach. In this paper we assess the reliability of a joint strategy using ERT and MALM techniques to identify the active root-zone of orange trees, undergoing full and deficit irrigation schemes. The study had the following objectives:

- Define a joint protocol that makes use of both ERT and MALM measurements to map the active root regions (in this case, for orange trees);
- Assess the reliability of the geophysical methods to explain different seasonal rooting pattern strategies for RWU processes (of orange trees) in presence of deficit and full irrigation;
- Integrate geophysical results with mass fluxes measurements in/out of the soil-plant continuum system.

## Results and Discussion

### Experimental site and irrigation scheduling

**Figure 1:**
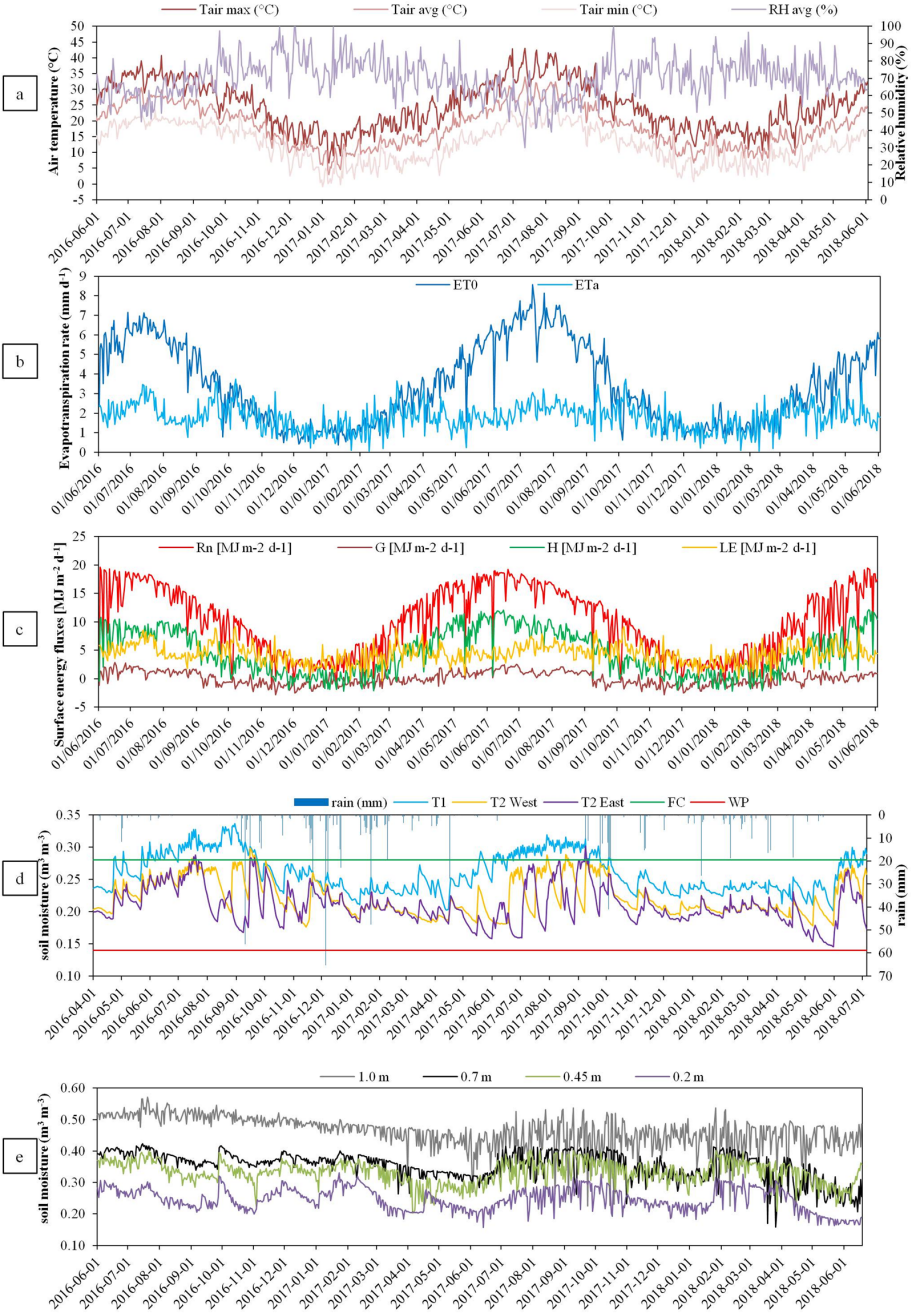
Seasonal ancillary monitoring at the experimental site: (**a**) climatic data (RH, relative humidity in %, Tair, air temperature); (**b**) evapotranspiration (ET0 and ETa, respectively the cumulative reference ET and the daily ET measured by eddy covariance (EC) system) rates measured by the EC system; (**c**) computed surface energy fluxes (Rn, net radiation; H, sensible heat flux; LE, latent heat flux, and G, soil heat flux; W.m^−2^); (**d**) SWC measured by FDR at 0.3 m at T1 and T2 (East and Ouest) below the surface, and corresponding rainfall events; and (**e**) SWC measured by TDR at different depths (in T1).

The experiments, including geophysical surveys (ERT and MALM), were carried out in an orange orchard managed by CREA-OFA^5^, located in Eastern Sicily, Italy (37° 20″N, 14° 53″E). The climate is semi-arid Mediterranean, with hot and dry summers. During the 2016–18 period, the maximum air temperature reached 43 °C, with mean relative humidity of 70% and the cumulative reference evapotranspiration (ET0) and rainfall were of 2498 mm and 972 mm, respectively (Fig. 1a,b) (data from Servizio Informativo Agrometeorologico Siciliano, SIAS).

Different DI strategies have been applied at this orange orchard ([Citrus sinensis (L.) Osbeck] cv “Tarocco Sciara” C1882 grafted on Carrizo citrange rootstock [Poncirus trifoliata (L.) Raf. × C. sinensis (L.) Osbeck]) since 2010. In this study we focus on two applied irrigation treatments: (i) full irrigation (T1), in which 11-year old trees are irrigated to replace 100% of the measured crop evapotranspiration (ETc); and (ii) Partial Root-zone Drying (PRD, T2), where trees receive 50% of measured ETc. Trees in T1 and T2 are drip irrigated using two surface pipelines (lied down at 0.3 m on either side of the trunks); each pipeline consists of six 4 L.h-1 drippers (spaced 0.6 m apart along the line) per tree. In T1, the two lines are placed next to each other, while in T2 irrigation was supplied alternatively to either side of the tree root-zone, while the other remains dry, with the system being switched fortnightly.

### ERT inversion and MALM data analysis

**Figure 2:**
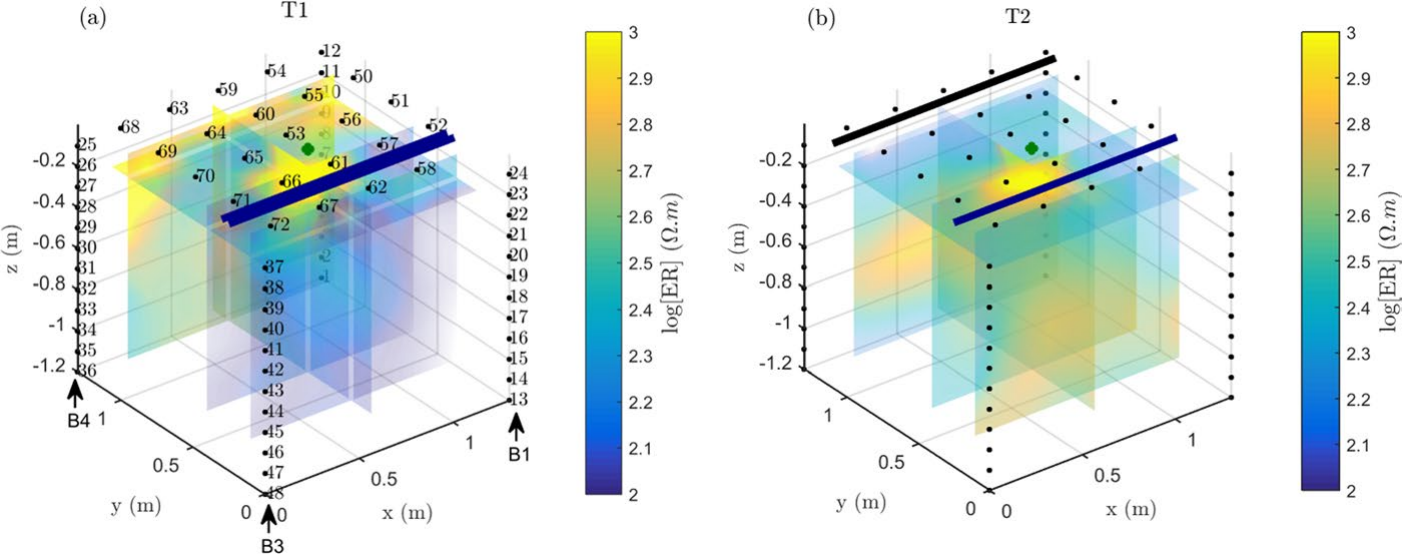
Electrical resistivity (ER) values from ERT inversion (in log scale) for (**a**) T1 and (**b**) T2; Numbering of the electrode and boreholes are displayed on the left figure; the two configurations are exactly the same. The green points represent the locations of the two trees, the blue lines show the active irrigation pipes, the black line (for T2) the non-active line at the time of the experiment (in the PRD irrigation regime).

At the end of the 2017 irrigation season we observed different patterns of electrical resistivity the root-zones in treatments T1 and T2 (Fig. 2), as a result (arguably) of the different irrigation strategies. Figure 2a shows clearly the impact of irrigation in T1, being the low resistivity anomaly correlated to the pipelines position (both on the same side of the tree, next to each other). Irrigation caused a uniform resistivity decrease along the vertical section below the dripper lines. Note that the resistivity contrast is relatively small, 10 and 20 ${\rm{\Omega }}\mathrm{.}m$. The electrical resistivity in T2, on the contrary, is much more homogeneous, as a result of the fortnightly irrigation alternation (Fig. 1d). For the irrigation pipeline active at the time of measurements (blue line, Fig. 2b), a resistivity decrease is observed. The other side (corresponding to the non-active irrigation pipeline, shown as a black line in Fig. 2b) had the time to dry after the previous irrigation period, so that the electrical resistivity decrease, corresponding to a wetter zone, is located at depth between 0.6 m and 1 m from the ground surface.

Both trees (in T1 and T2) show a similar pattern of higher resistivity near the tree trunk, for a horizontal diameter of about 0.2 m, and a vertical extent of about 0.4 m (Fig. 2). Arguably, this anomaly corresponds to the bulk of the woody roots, with a primary structural role, similar in both cases as the two plants have similar age and size. Note that this resistive anomaly does not allow, however, to estimate the extent of the active root system.

**Figure 3:**
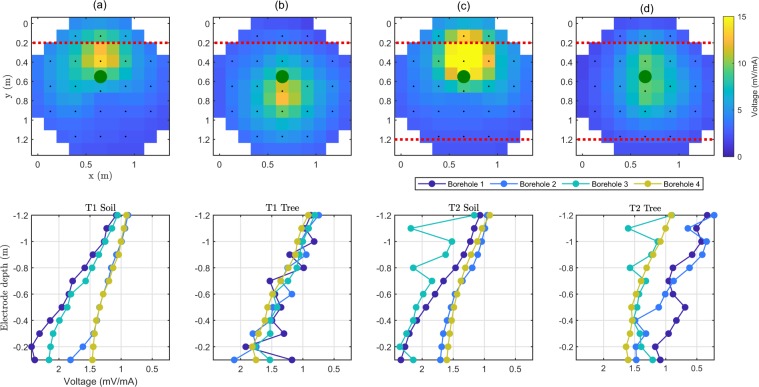
MALM data. Top row: from left to right, spatial distribution of the normalized voltage (in V/A) measured at surface electrodes for the soil injection (**a**) and stem injection (**b**) in T1; for the soil injection (**c**) and stem injection (**d**) in T2. The green points represent the locations of the two trees. Bottom row: same as for the top row, but profile data from the boreholes electrodes.

Figure 3 shows the MALM data (voltage normalized over the injection current) acquired at the same time on both treatments (T1 and T2) using soil and stem injection, for a total of 4 maps (produced using surface electrodes) and 4 profiles as a function of depth (produced using the borehole electrodes). These images show clearly that the system response is very different for the two plants, and is also very different in case the current is injected in the stem or in the soil next to the stem. The latter fact shows that the root system somehow conveys current in a different manner than the soil per se, and this fact supports the main assumption behind this study i.e. that the root system allows electrical current to emerge in the soil at locations where the roots themselves are in close contact with the soil, i.e. most likely where the roots uptake water from the soil (see section Methods). In T1 (full irrigation - control) soil injection produces a voltage anomaly with a strong asymmetry in one direction, arguably caused by a heterogeneous resistivity distribution in the subsoil. A similar asymmetry is observed, for injection in the soil, also in T2. Note that the normalized voltage values due to soil injection are substantially different in T1 and T2 (about a factor of 2 between the two maps). The resistivity differences between T1 and T2 (Fig. 2) justifies this difference, as a smaller electrical conductivity at depth, such as in T1, caused by a larger water content across the profile, in turn caused by a larger irrigation rate. In addition, note that the voltage maximum, in the interpolated map, is slightly shifted with respect to its true location, that must be in correspondence of the injection electrode. This is purely an effect of interpolation, as the voltage electrodes are evenly spaced at the surface, with no electrode close to the injecting one. For injection in the soil, there is no physical process that may cause this asymmetry considering that we used for the injection an electrode of small diameter and length (buried to 2 cm depth). In this case the hypothesis of a perfectly punctual current source is valid.

When current is injected into the stem, (Fig. 3b for T1 and Fig. 3d for T2) the voltage distribution is very different to the one caused by the injection made in soil, next to the stem. In T1 (Fig. 3b) the corresponding anomaly is more spatially diffuse than for soil injection, while in T2 (Fig. 3d) the anomaly extent is even larger. Some clear differences are also noticeable in the borehole electrode profiles (Fig. 3), not necessarily with a straightforward interpretation. In all cases, the MALM current injection into the stem produces, at surface electrodes, a lower signal than in the case of direct soil injection. For the surface electrodes in T2 (Fig. 3a) the mean voltage of the anomaly voltage for soil injection was slightly higher (+2 mV/mA) than for stem injection (Fig. 3b), while in T1 (Fig. 3c), the mean voltage of the surface electrode anomaly for soil injection was much higher (+15 mV/mA) than for stem injection (Fig. 3d). This is another indication that the stem-roots system conveys current preferably at depth, while the injection in the soil keeps the maximum voltage at the surface, as expected from basic geo-electrical theory. Interestingly, unlike in the case of surface electrodes, the voltage measured by borehole electrodes is sensitive to the irrigation strategy, and more precisely to the irrigation pipeline position. The voltage distribution along boreholes is clearly different in T2 than in T1 (Fig. 3). This is a clear effect of the resistivity distribution differences between T1 and T2 (Fig. 2) which in turn is a function of the soil moisture content differences, caused by the different irrigation strategies. Details can be appreciated in Fig. 3.

### MALM inversion results

**Figure 4:**
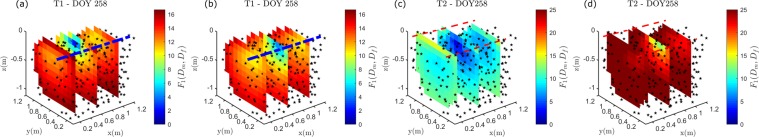
MALM inversion results. The 3D images represent the spatial distribution of score F1 given to each single candidate current location (see step 2, section Methods). Particularly in the case of the deficit irrigation experiment (**c**,**d**), the F1 pattern is totally different for soil (**c**) and stem current injections (**d**), thus indicating that the root distribution is likely deep in the soil. Not so for the full irrigation case, where the two images (**a**,**b**) are not so different, indicating that roots are not pushed to developing at depth, as water is readily available at the surface.

MALM inversion, as we implemented it, is a two-step process. Figure 4 shows primarily the results of step 2 as 3D contours plots of score F1 (see section Methods - Data processing (DP)). The figure shows clearly that in the case of deficit irrigation (PRD - top row), the distribution of likely current source locations, when the current is injected in the stem, is very different from the corresponding distribution when current is injected in the soil. Such a strong difference is not observed in the case of full irrigation (Fig. 4, bottom row). This is an indication that in T1 (full irrigation) the active roots distribution is very different from the T2 case (deficit irrigation). In particular, if the key assumption (see section Method-DP) holds, the evidence shown in Fig. 4 indicates that the active root system in T2 is deeper than in T1. And in T1 (full irrigation) the root system is shallow enough not to add any component to the effect of injecting current directly in the soil rather than in the stem. This may be indicating that, in T1, roots are not pushed to developing at depth, as enough water is readily available at the surface.

**Figure 5:**
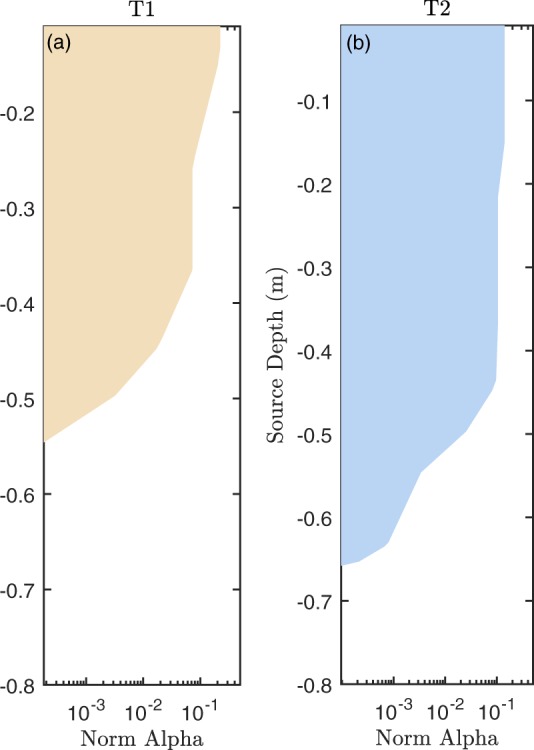
MALM inversion results summarized in terms of average fraction of current source strengths (|α|) as a function of depth. The profiles confirm that in the PRD case (**b**) roots tend to develop deeper in search for soil water at a depth compared to the full irrigation case (**a**).

The actual inversion for source location injected currents (point 3 in section Methods-DP) leads to results not different from the preliminary inversion just discussed:

- For both T1 and T2, in the case of current injection in the soil, the inversion results indicate that the current density is very high close to the soil injection point (details not shown): this testifies of the robustness of the inversion process;
- In the case of stem injection, on the other hand, a difference between T1 (full irrigation) and T2 (deficit irrigation) is apparent. Figure 5 shows also the profiles of estimated total source current percentage as a function of depth: for T2 the extent of sources is larger at depth, confirming that the active root system is likely to be deeper in the case of deficit irrigation.

## Discussion

The origins of the variations of electrical resistivity measured in rooted area are still not fully understood but literature mainly reports that there are predominant effect of RWU on soil moisture content over time rather than the ligneous nature of large roots. Conversely, MALM appears to be much more sensitive to the root system itself. The estimated rooting depth and active root system respectively inferred from the ERT and MALM results is in the range of expected values for irrigated crops (e.g. Introduction). It also appears that the lateral extent of the root system may have been influenced by the position of the dripper lines since even after correction of heterogeneous resistivity distribution in the subsoil, the spatial distribution of score F1 still remains asymmetric (Fig. 4a). In that particular case, reducing the problem to a root length density profile (i.e. linear, non linear or exponential, see Fig. 5) as it is done in numerous RWU models, may potentially lead to a biased estimation of the hydrological model parameters. Although our results are promising, MALM reliability to explain different seasonal rooting pattern strategies remains a challenging task. The physiological stage of the plant has numerous implication on the geophysical measurements. How the current propagates through the roots and is released to the soil depends also on the root electrical conductivity, which in turn is related to roots hydraulic conductance distribution which also controls RWU processes.

In light of the biological and physiological assumptions, we suggest that differences observed at our site in MALM response between the two investigated trees is a consequence of both short and long term effects of structural and physiological changes. The PRD tree (T2) was subjected to drought as evidenced by a soil water content (at 0.3 m depth) constantly below the field capacity level and almost reaching the wilting point during the 2017 summer. The rooting strategy to adapt to drought may have caused the formation of deeper roots. This assumption is supported by the MALM result showing a deeper zone where the root are active for T2 (PRD) but not for T1. Nevertheless, Vanella *et al*.^3^ who used ERT monitoring on the same trees T1 and T2, stated that shallow and deep root zones both appear to be active during different times of the growing season, depending on water availability. Accordingly, the way the root system can be seen via the MALM acquisition is likely to differ seasonally as a consequence of the changing distribution of active roots which ultimately determine the preferential current path propagation. Electrical properties of roots are not only affected by structure but also by physiological processes as demonstrated by^61,62^ on crop root suffering of nutrient deprivation using electrical impedance tomography measurements. In our study, although the root-to-shoot ratio may have tended to increase under drought conditions, the biomass of fine roots could have been reduced as a consequence of reduced transpiration and respiration rates. Reduction of root tip frequency, along with adjustment of their hydraulic conductivity, suggested that the current release is likely to be affected. As reported by^63^ the lifespan of fine roots growing in dry soil might be reduced in the absence of hydraulic redistribution. However, in our study the application of a PRD irrigation might be long enough to allow for a redistribution of water from wetter soil to dryer soil, thus maintaining the root system stable in time^64^. Overall, the below-ground responses to irrigation are less evident than the more rapid adaptations aboveground^65^. It is also difficult to produce an overall image of the Soil-Plant-Atmosphere-Continuum behaviour at the time frame of this experiment.

## Conclusion

This study presents a successful use of two well-established geo-electrical methods in a novel configuration, where the information content of both is fully exploited in a joint manner. The results we present relevant to a field study indicate that this joint approach can see differences in the plant rooting systems caused, e.g., by different irrigation strategies. Note that the sensitivity of ERT to soil moisture content is not exploited in the presented approach. This, however, can be a further step for a complete exploitation of measurement information. Also, the effect of soil moisture content changes on MALM response, as a proxy of changing rooting strategies, is also to be exploited. Thus the presented approach can be the basis for further exciting developments.

## Methods

### Long period micrometeorological and soil water status measurements

In 2016, an eddy covariance (EC) system was installed at the experimental orange orchard, using a 7 m tall micrometeorological tower (about two times the canopy height). The micrometeorological system allows measurements of mass and energy fluxes exchanges (Rn, net radiation; H, sensible heat flux; LE, latent heat flux, and G, soil heat flux; W.m^−2^) at a half-hourly rate (Fig. 1b,c). Rn is the dominant surface energy balance component, driving the energy budget. In T1 and T2, SWC (m^3^m^−3^) was monitored using ECH2O probes (Decagon, Inc.), placed at 0.3 m depth. In T2, the probes are located at both sides the trunk (Fig. 1d). In T1, a time-domain reflectometer (TDR-100 Campbell Scientific) was also installed to measure SWC at different depths (0.2, 0.45, 0.7 and 1.0 m) (Fig. 1e). Soil hydraulic parameters, such as field capacity (FC, 0.28 m^3^m^−3^) and permanent wilting point (WP, 0.14 m^3^m^−3^) were determined by laboratory analyses of the soil samples, having a sandy loam texture^1,66^. During the 2016–2018 irrigation period, daily SWC (at 0.3 m of depth) ranged within FC and WP at both T1 and T2, with average values of about 0.22(±0.03) and 0.26 (±0.03) m^3^m^−3^, respectively. Minimum daily SWC was recorded on the T2 eastern side (0.15 m^3^m^−3^).

### Small scale 3-D ERT and MALM acquisition, processing and interpretation

#### Data collection

In this paper we present the results of a specific experiment conducted in September 2017 (DOY 258). For both monitored trees (in T1 and T2), both ERT and MALM acquisitions started before irrigation. Figure 2 shows the spatial setup of the borehole and surface electrodes used for ERT and MALM acquisitions at both trees (T1 and T2). The small electrode spacing and a suitable acquisition protocol (see below) ensure a resolution sufficient to image the effect of roots on soil water content, and possibly also image the roots per se (see details below). Three datasets were collected during the experiment, at a short time difference from each other (minutes): (i) a 3D ERT dataset, based on a skip 2 complete dipole-dipole configuration, composed of 5098 measurement points, with full reciprocal acquisition (see e.g.^67^); (ii) a 3D MALM dataset using a pole-pole configuration with a fixed current electrode (A) inserted into the tree trunk, a remote current return electrode (B), a reference voltage remote electrode (N) and each remaining electrode (69) serving as voltage measuring points; (iii) a 3D MALM control dataset for which the current electrode A was placed in the soil, close to the trunk. For all acquisitions, we used an IRIS Instruments Syscal Pro resistivity meter, with an injection time of 250 ms, a maximum injection voltage of 800 V, and a minimum voltage reception value equal to 50 mV.

#### Data processing

The goal of data processing is to exploit the information content of both ERT and MALM in a joint approach, aimed at imaging both SWC changes caused by RWU, and the location of active roots per se. We performed a three-step data processing/inversion, similar to the one described by^60^, involving both ERT and MALM data:

- Step 1: inversion of the resistance data collected using the 3D ERT scheme (we used the R3t code -^68^). The inverse solution is based on a regularized objective function combined with weighted least squares (an Occam’s type solution) as presented e.g. by^69^. Prior to inversion, data quality was assessed using a 10% threshold on reciprocal measurements (see e.g.^67^, for details);
- Step 2: a preliminary step to analyse MALM data: we produce a forward modelling of the voltage distribution generated by individual point current sources placed in the soil, each representing the possible location of current released from the plant roots. We hypothesized approximately 500 locations for current sources, uniformly distributed in space. The forward simulation takes into account the electrical resistivity distribution as obtained by ERT inversion (see step above). The goal of this step is to select “plausible” locations for single roots (conceived as current sources, i.e. points where current flows from the root into the soil in the MALM experiment), to be then used as starting points for a regularized search of the overall sources distribution (see step below). Each single current location is given a score (named F1) depending on how well that single source manages, alone, to explain the entire observed MALM voltage distribution.
- Step 3: a proper inversion of MALM data (voltage) in terms of current source intensities and locations in the soil. The inversion takes advantage of the plausible locations identified in step 2: the locations are taken into account only if their score is above a given threshold, thus restricting the region of candidates and regularizing the inversion. The inversion of current source is based on the minimization of the overall discrepancy between measured and simulated MALM data, where the unknowns to be identified are the fractions of the total current to be assigned to each “plausible” source location (in the sense of step 2).

Note that steps 2 and 3, which overall represent the inversion of MALM data, are applied to both experiments involving current injection in the stem and current injection in the soil next to the stem, in order to appreciate the resulting differences.

#### Data interpretation assumption

We interpret the inversion results in terms of spatial distribution of the active root system. This entails that one main assumption holds, i.e. that thanks to the high electrical conductivity of the root cells, seat of ions and water exchange with the plant upper part, and constituting a water path continuum, the root system is acting as a conductive body which conducts the current from the tree trunk (where it is injected in the MALM experiment) to the roots. The current is then release to the soil by the root tips or at locations where hair roots penetrate the soil. The observation of the strong differences between the MALM data and the control MALM acquired with the soil injection close to the trunk contributes to validate this assumption.
